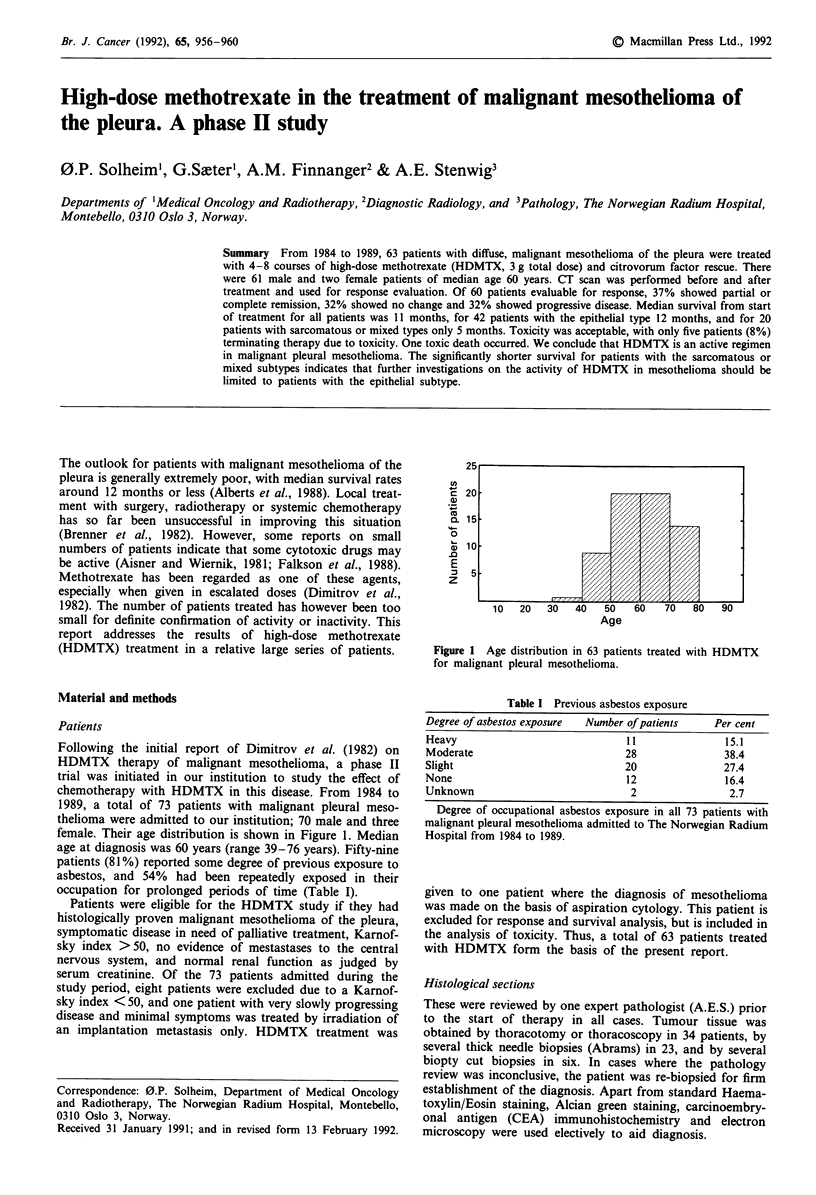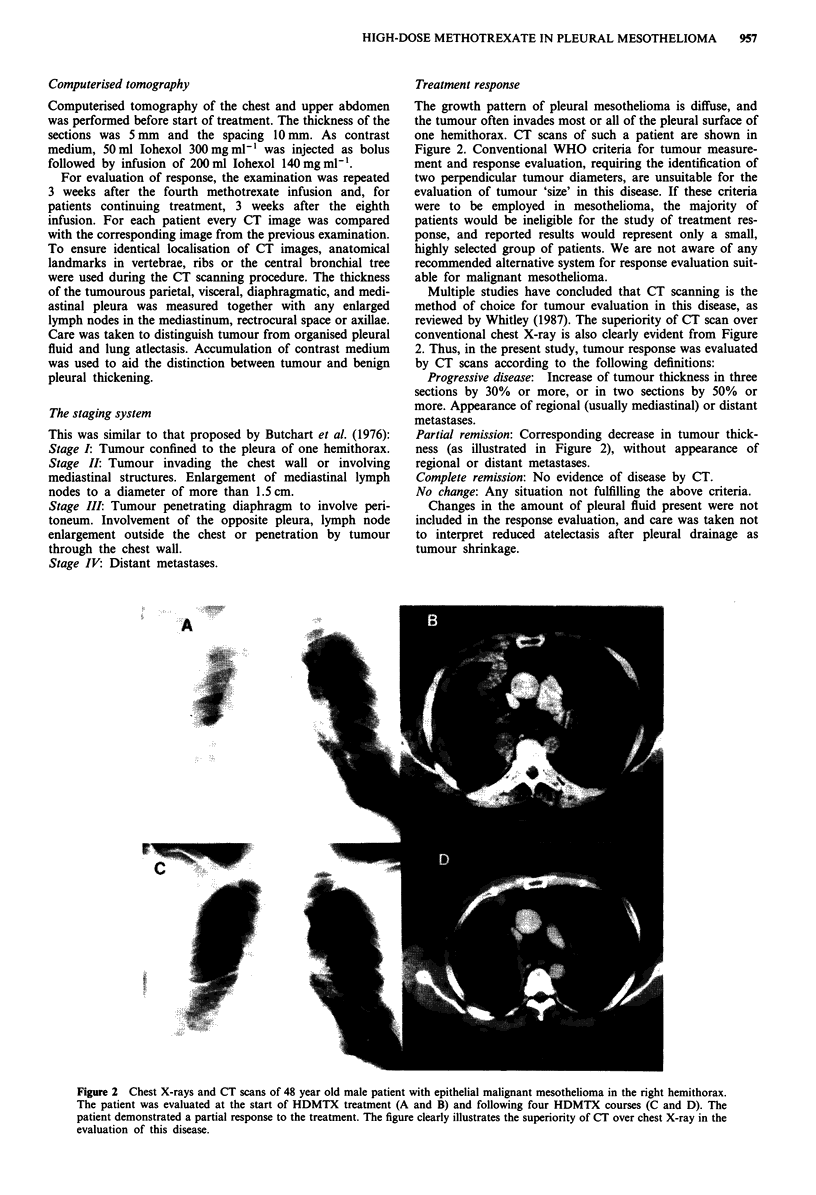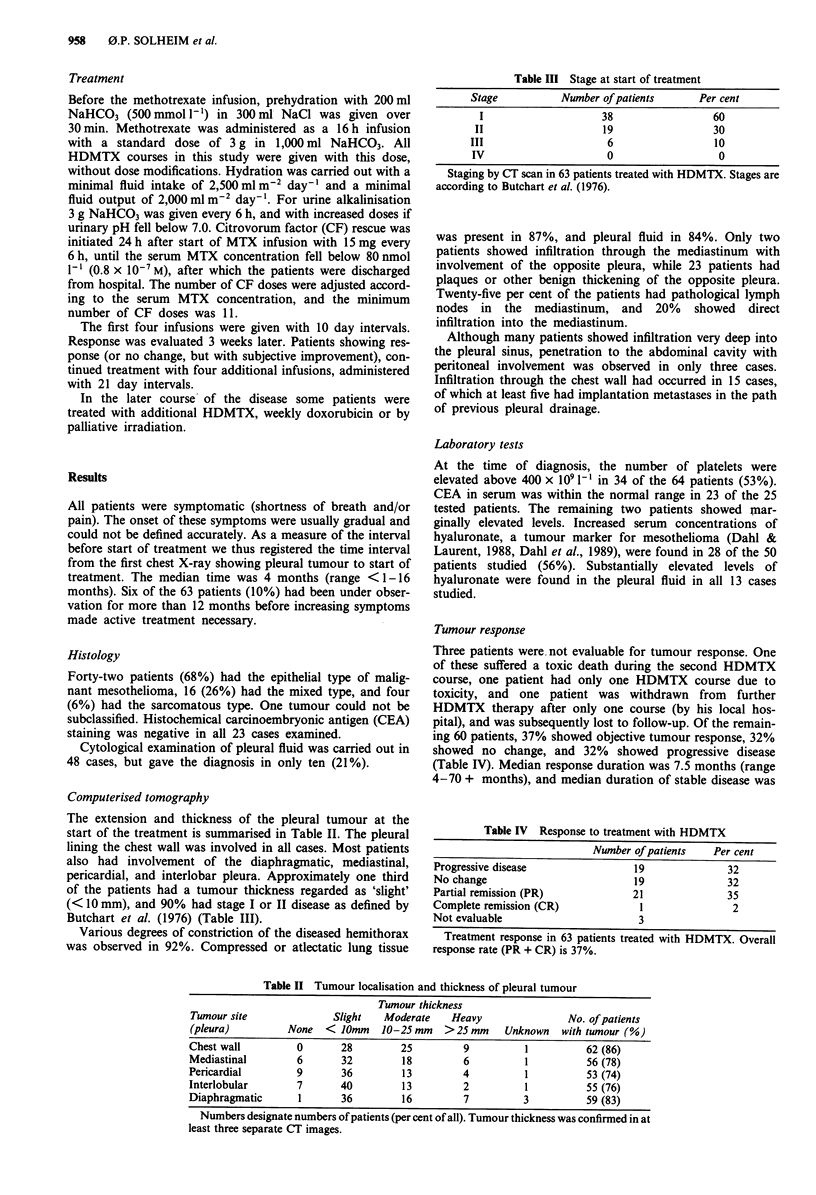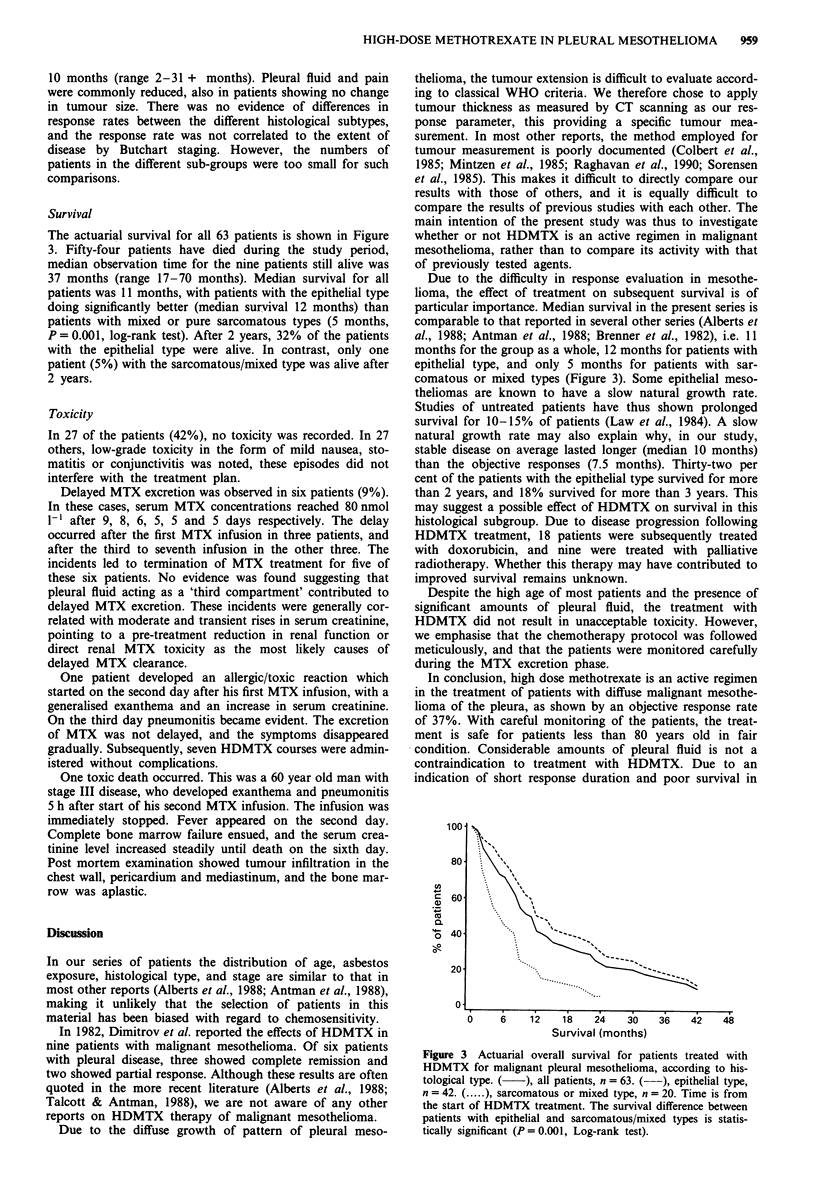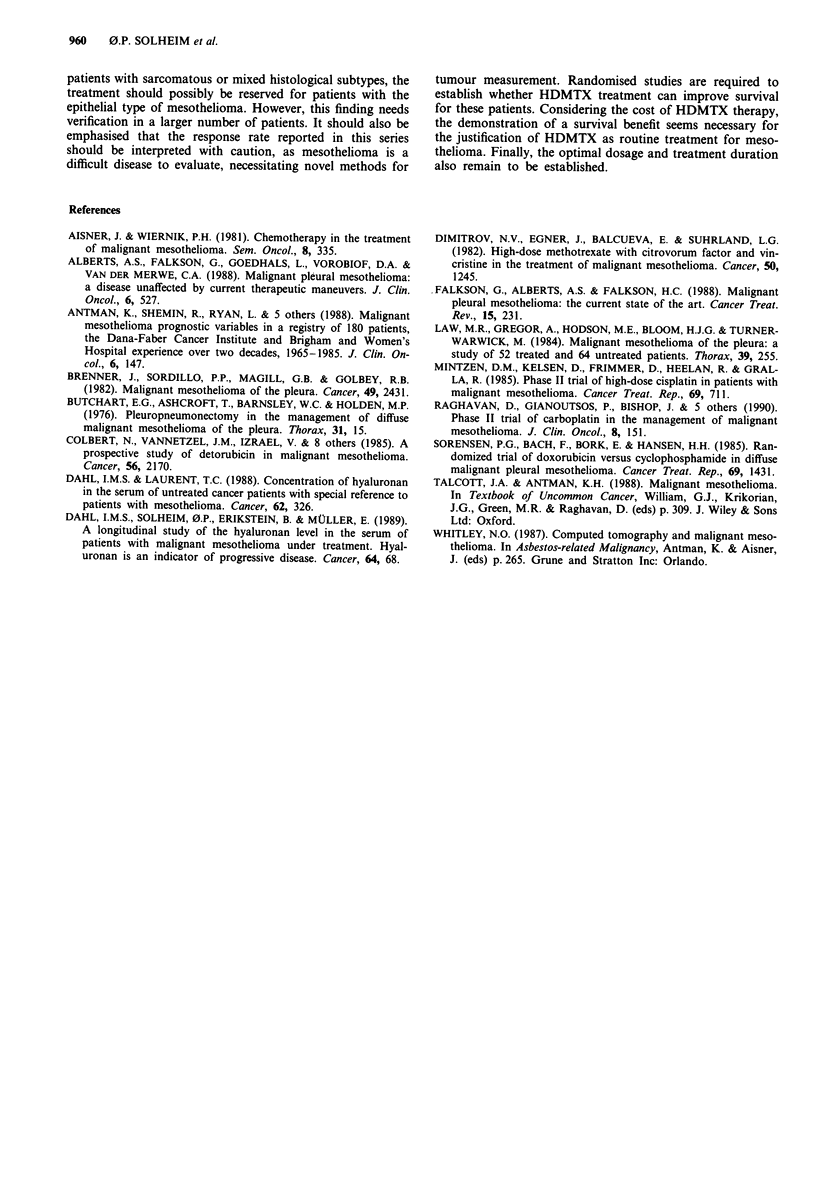# High-dose methotrexate in the treatment of malignant mesothelioma of the pleura. A phase II study.

**DOI:** 10.1038/bjc.1992.200

**Published:** 1992-06

**Authors:** O. P. Solheim, G. Saeter, A. M. Finnanger, A. E. Stenwig

**Affiliations:** Department of Medical Oncology and Radiotherapy, Norwegian Radium Hospital, Montebello, Oslo.

## Abstract

**Images:**


Br. J. Cancer (1992), 65, 956-960 © Macmillan Press Ltd., 1992

### High-dose methotrexate in the treatment of malignant mesothelioma of the pleura. A phase II study

Ø.P. Solheim[1], G.Sæter[1], A.M. Finnanger[2] & A.E. Stenwig[3]

*Departments of [1]Medical Oncology and Radiotherapy, [2]Diagnostic Radiology, and [3]Pathology, The Norwegian Radium Hospital, Montebello, 0310 Oslo 3, Norway.*

**Summary** From 1984 to 1989, 63 patients with diffuse, malignant mesothelioma of the pleura were treated with 4–8 courses of high-dose methotrexate (HDMTX, 3 g total dose) and citrovorum factor rescue. There were 61 male and two female patients of median age 60 years. CT scan was performed before and after treatment and used for response evaluation. Of 60 patients evaluable for response, 37% showed partial or complete remission, 32% showed no change and 32% showed progressive disease. Median survival from start of treatment for all patients was 11 months, for 42 patients with the epithelial type 12 months, and for 20 patients with sarcomatous or mixed types only 5 months. Toxicity was acceptable, with only five patients (8%) terminating therapy due to toxicity. One toxic death occurred. We conclude that HDMTX is an active regimen in malignant pleural mesothelioma. The significantly shorter survival for patients with the sarcomatous or mixed subtypes indicates that further investigations on the activity of HDMTX in mesothelioma should be limited to patients with the epithelial subtype.

The outlook for patients with malignant mesothelioma of the pleura is generally extremely poor, with median survival rates around 12 months or less (Alberts *et al.*, 1988). Local treatment with surgery, radiotherapy or systemic chemotherapy has so far been unsuccessful in improving this situation (Brenner *et al.*, 1982). However, some reports on small numbers of patients indicate that some cytotoxic drugs may be active (Aisner and Wiernik, 1981; Falkson *et al.*, 1988). Methotrexate has been regarded as one of these agents, especially when given in escalated doses (Dimitrov *et al.*, 1982). The number of patients treated has however been too small for definite confirmation of activity or inactivity. This report addresses the results of high-dose methotrexate (HDMTX) treatment in a relative large series of patients.

**Figure 1** Age distribution in 63 patients treated with HDMTX for malignant pleural mesothelioma.

### Material and methods

### *Patients*

Following the initial report of Dimitrov *et al.* (1982) on HDMTX therapy of malignant mesothelioma, a phase II trial was initiated in our institution to study the effect of chemotherapy with HDMTX in this disease. From 1984 to 1989, a total of 73 patients with malignant pleural mesothelioma were admitted to our institution; 70 male and three female. Their age distribution is shown in Figure 1. Median age at diagnosis was 60 years (range 39–76 years). Fifty-nine patients (81%) reported some degree of previous exposure to asbestos, and 54% had been repeatedly exposed in their occupation for prolonged periods of time (Table I).

**Table I** Previous asbestos exposure

| *Degree of asbestos exposure* | *Number of patients* | *Per cent* |
|---|---|---|
| Heavy | 11 | 15.1 |
| Moderate | 28 | 38.4 |
| Slight | 20 | 27.4 |
| None | 12 | 16.4 |
| Unknown | 2 | 2.7 |

Degree of occupational asbestos exposure in all 73 patients with malignant pleural mesothelioma admitted to The Norwegian Radium Hospital from 1984 to 1989.

Patients were eligible for the HDMTX study if they had histologically proven malignant mesothelioma of the pleura, symptomatic disease in need of palliative treatment, Karnofsky index >50, no evidence of mestastases to the central nervous system, and normal renal function as judged by serum creatinine. Of the 73 patients admitted during the study period, eight patients were excluded due to a Karnofsky index <50, and one patient with very slowly progressing disease and minimal symptoms was treated by irradiation of an implantation metastasis only. HDMTX treatment was given to one patient where the diagnosis of mesothelioma was made on the basis of aspiration cytology. This patient is excluded for response and survival analysis, but is included in the analysis of toxicity. Thus, a total of 63 patients treated with HDMTX form the basis of the present report.

### *Histological sections*

These were reviewed by one expert pathologist (A.E.S.) prior to the start of therapy in all cases. Tumour tissue was obtained by thoracotomy or thoracoscopy in 34 patients, by several thick needle biopsies (Abrams) in 23, and by several biopty cut biopsies in six. In cases where the pathology review was inconclusive, the patient was re-biopsied for firm establishment of the diagnosis. Apart from standard Haematoxylin/Eosin staining, Alcian green staining, carcinoembryonal antigen (CEA) immunohistochemistry and electron microscopy were used electively to aid diagnosis.

Correspondence: Ø.P. Solheim, Department of Medical Oncology and Radiotherapy, The Norwegian Radium Hospital, Montebello, 0310 Oslo 3, Norway.
Received 31 January 1991; and in revised form 13 February 1992.

*Computerised tomography*

Computerised tomography of the chest and upper abdomen was performed before start of treatment. The thickness of the sections was 5 mm and the spacing 10 mm. As contrast medium, 50 ml Iohexol 300 mg $ml^{-1}$ was injected as bolus followed by infusion of 200 ml Iohexol 140 mg $ml^{-1}$.

For evaluation of response, the examination was repeated 3 weeks after the fourth methotrexate infusion and, for patients continuing treatment, 3 weeks after the eighth infusion. For each patient every CT image was compared with the corresponding image from the previous examination. To ensure identical localisation of CT images, anatomical landmarks in vertebrae, ribs or the central bronchial tree were used during the CT scanning procedure. The thickness of the tumourous parietal, visceral, diaphragmatic, and mediastinal pleura was measured together with any enlarged lymph nodes in the mediastinum, rectrocural space or axillae. Care was taken to distinguish tumour from organised pleural fluid and lung atlectasis. Accumulation of contrast medium was used to aid the distinction between tumour and benign pleural thickening.

*The staging system*

This was similar to that proposed by Butchart *et al.* (1976):
*Stage I*: Tumour confined to the pleura of one hemithorax.
*Stage II*: Tumour invading the chest wall or involving mediastinal structures. Enlargement of mediastinal lymph nodes to a diameter of more than 1.5 cm.
*Stage III*: Tumour penetrating diaphragm to involve peritoneum. Involvement of the opposite pleura, lymph node enlargement outside the chest or penetration by tumour through the chest wall.
*Stage IV*: Distant metastases.

*Treatment response*

The growth pattern of pleural mesothelioma is diffuse, and the tumour often invades most or all of the pleural surface of one hemithorax. CT scans of such a patient are shown in Figure 2. Conventional WHO criteria for tumour measurement and response evaluation, requiring the identification of two perpendicular tumour diameters, are unsuitable for the evaluation of tumour 'size' in this disease. If these criteria were to be employed in mesothelioma, the majority of patients would be ineligible for the study of treatment response, and reported results would represent only a small, highly selected group of patients. We are not aware of any recommended alternative system for response evaluation suitable for malignant mesothelioma.

Multiple studies have concluded that CT scanning is the method of choice for tumour evaluation in this disease, as reviewed by Whitley (1987). The superiority of CT scan over conventional chest X-ray is also clearly evident from Figure 2. Thus, in the present study, tumour response was evaluated by CT scans according to the following definitions:

*Progressive disease*: Increase of tumour thickness in three sections by 30% or more, or in two sections by 50% or more. Appearance of regional (usually mediastinal) or distant metastases.
*Partial remission*: Corresponding decrease in tumour thickness (as illustrated in Figure 2), without appearance of regional or distant metastases.
*Complete remission*: No evidence of disease by CT.
*No change*: Any situation not fulfilling the above criteria.

Changes in the amount of pleural fluid present were not included in the response evaluation, and care was taken not to interpret reduced atelectasis after pleural drainage as tumour shrinkage.

**Figure 2** Chest X-rays and CT scans of 48 year old male patient with epithelial malignant mesothelioma in the right hemithorax. The patient was evaluated at the start of HDMTX treatment (A and B) and following four HDMTX courses (C and D). The patient demonstrated a partial response to the treatment. The figure clearly illustrates the superiority of CT over chest X-ray in the evaluation of this disease.

*Treatment*

Before the methotrexate infusion, prehydration with 200 ml $NaHCO_3$ (500 mmol $l^{-1}$) in 300 ml NaCl was given over 30 min. Methotrexate was administered as a 16 h infusion with a standard dose of 3 g in 1,000 ml $NaHCO_3$. All HDMTX courses in this study were given with this dose, without dose modifications. Hydration was carried out with a minimal fluid intake of 2,500 ml $m^{-2}$ $day^{-1}$ and a minimal fluid output of 2,000 ml $m^{-2}$ $day^{-1}$. For urine alkalinisation 3 g $NaHCO_3$ was given every 6 h, and with increased doses if urinary pH fell below 7.0. Citrovorum factor (CF) rescue was initiated 24 h after start of MTX infusion with 15 mg every 6 h, until the serum MTX concentration fell below 80 nmol $l^{-1}$ ($0.8 \times 10^{-7}$ M), after which the patients were discharged from hospital. The number of CF doses were adjusted according to the serum MTX concentration, and the minimum number of CF doses was 11.

The first four infusions were given with 10 day intervals. Response was evaluated 3 weeks later. Patients showing response (or no change, but with subjective improvement), continued treatment with four additional infusions, administered with 21 day intervals.

In the later course of the disease some patients were treated with additional HDMTX, weekly doxorubicin or by palliative irradiation.

### Results

All patients were symptomatic (shortness of breath and/or pain). The onset of these symptoms were usually gradual and could not be defined accurately. As a measure of the interval before start of treatment we thus registered the time interval from the first chest X-ray showing pleural tumour to start of treatment. The median time was 4 months (range $<1-16$ months). Six of the 63 patients (10%) had been under observation for more than 12 months before increasing symptoms made active treatment necessary.

*Histology*

Forty-two patients (68%) had the epithelial type of malignant mesothelioma, 16 (26%) had the mixed type, and four (6%) had the sarcomatous type. One tumour could not be subclassified. Histochemical carcinoembryonic antigen (CEA) staining was negative in all 23 cases examined.

Cytological examination of pleural fluid was carried out in 48 cases, but gave the diagnosis in only ten (21%).

*Computerised tomography*

The extension and thickness of the pleural tumour at the start of the treatment is summarised in Table II. The pleural lining the chest wall was involved in all cases. Most patients also had involvement of the diaphragmatic, mediastinal, pericardial, and interlobar pleura. Approximately one third of the patients had a tumour thickness regarded as 'slight' ($<10$ mm), and 90% had stage I or II disease as defined by Butchart *et al.* (1976) (Table III).

Various degrees of constriction of the diseased hemithorax was observed in 92%. Compressed or atlectatic lung tissue was present in 87%, and pleural fluid in 84%. Only two patients showed infiltration through the mediastinum with involvement of the opposite pleura, while 23 patients had plaques or other benign thickening of the opposite pleura. Twenty-five per cent of the patients had pathological lymph nodes in the mediastinum, and 20% showed direct infiltration into the mediastinum.

Although many patients showed infiltration very deep into the pleural sinus, penetration to the abdominal cavity with peritoneal involvement was observed in only three cases. Infiltration through the chest wall had occurred in 15 cases, of which at least five had implantation metastases in the path of previous pleural drainage.

**Table III** Stage at start of treatment

| *Stage* | *Number of patients* | *Per cent* |
|---|---|---|
| I | 38 | 60 |
| II | 19 | 30 |
| III | 6 | 10 |
| IV | 0 | 0 |

Staging by CT scan in 63 patients treated with HDMTX. Stages are according to Butchart *et al.* (1976).

*Laboratory tests*

At the time of diagnosis, the number of platelets were elevated above $400 \times 10^9$ $l^{-1}$ in 34 of the 64 patients (53%). CEA in serum was within the normal range in 23 of the 25 tested patients. The remaining two patients showed marginally elevated levels. Increased serum concentrations of hyaluronate, a tumour marker for mesothelioma (Dahl & Laurent, 1988, Dahl *et al.*, 1989), were found in 28 of the 50 patients studied (56%). Substantially elevated levels of hyaluronate were found in the pleural fluid in all 13 cases studied.

*Tumour response*

Three patients were not evaluable for tumour response. One of these suffered a toxic death during the second HDMTX course, one patient had only one HDMTX course due to toxicity, and one patient was withdrawn from further HDMTX therapy after only one course (by his local hospital), and was subsequently lost to follow-up. Of the remaining 60 patients, 37% showed objective tumour response, 32% showed no change, and 32% showed progressive disease (Table IV). Median response duration was 7.5 months (range 4–70 + months), and median duration of stable disease was

**Table IV** Response to treatment with HDMTX

| | *Number of patients* | *Per cent* |
|---|---|---|
| Progressive disease | 19 | 32 |
| No change | 19 | 32 |
| Partial remission (PR) | 21 | 35 |
| Complete remission (CR) | 1 | 2 |
| Not evaluable | 3 | |

Treatment response in 63 patients treated with HDMTX. Overall response rate (PR + CR) is 37%.

**Table II** Tumour localisation and thickness of pleural tumour

| *Tumour site (pleura)* | *None* | *Tumour thickness* *Slight* $<$ *10mm* | *Moderate 10–25 mm* | *Heavy* $>$ *25 mm* | *Unknown* | *No. of patients with tumour (%)* |
|---|---|---|---|---|---|---|
| Chest wall | 0 | 28 | 25 | 9 | 1 | 62 (86) |
| Mediastinal | 6 | 32 | 18 | 6 | 1 | 56 (78) |
| Pericardial | 9 | 36 | 13 | 4 | 1 | 53 (74) |
| Interlobular | 7 | 40 | 13 | 2 | 1 | 55 (76) |
| Diaphragmatic | 1 | 36 | 16 | 7 | 3 | 59 (83) |

Numbers designate numbers of patients (per cent of all). Tumour thickness was confirmed in at least three separate CT images.

10 months (range 2–31 + months). Pleural fluid and pain were commonly reduced, also in patients showing no change in tumour size. There was no evidence of differences in response rates between the different histological subtypes, and the response rate was not correlated to the extent of disease by Butchart staging. However, the numbers of patients in the different sub-groups were too small for such comparisons.

*Survival*

The actuarial survival for all 63 patients is shown in Figure 3. Fifty-four patients have died during the study period, median observation time for the nine patients still alive was 37 months (range 17–70 months). Median survival for all patients was 11 months, with patients with the epithelial type doing significantly better (median survival 12 months) than patients with mixed or pure sarcomatous types (5 months, $P = 0.001$, log-rank test). After 2 years, 32% of the patients with the epithelial type were alive. In contrast, only one patient (5%) with the sarcomatous/mixed type was alive after 2 years.

*Toxicity*

In 27 of the patients (42%), no toxicity was recorded. In 27 others, low-grade toxicity in the form of mild nausea, stomatitis or conjunctivitis was noted, these episodes did not interfere with the treatment plan.

Delayed MTX excretion was observed in six patients (9%). In these cases, serum MTX concentrations reached 80 nmol $l^{-1}$ after 9, 8, 6, 5, 5 and 5 days respectively. The delay occurred after the first MTX infusion in three patients, and after the third to seventh infusion in the other three. The incidents led to termination of MTX treatment for five of these six patients. No evidence was found suggesting that pleural fluid acting as a 'third compartment' contributed to delayed MTX excretion. These incidents were generally correlated with moderate and transient rises in serum creatinine, pointing to a pre-treatment reduction in renal function or direct renal MTX toxicity as the most likely causes of delayed MTX clearance.

One patient developed an allergic/toxic reaction which started on the second day after his first MTX infusion, with a generalised exanthema and an increase in serum creatinine. On the third day pneumonitis became evident. The excretion of MTX was not delayed, and the symptoms disappeared gradually. Subsequently, seven HDMTX courses were administered without complications.

One toxic death occurred. This was a 60 year old man with stage III disease, who developed exanthema and pneumonitis 5 h after start of his second MTX infusion. The infusion was immediately stopped. Fever appeared on the second day. Complete bone marrow failure ensued, and the serum creatinine level increased steadily until death on the sixth day. Post mortem examination showed tumour infiltration in the chest wall, pericardium and mediastinum, and the bone marrow was aplastic.

### Discussion

In our series of patients the distribution of age, asbestos exposure, histological type, and stage are similar to that in most other reports (Alberts *et al.*, 1988; Antman *et al.*, 1988), making it unlikely that the selection of patients in this material has been biased with regard to chemosensitivity.

In 1982, Dimitrov *et al.* reported the effects of HDMTX in nine patients with malignant mesothelioma. Of six patients with pleural disease, three showed complete remission and two showed partial response. Although these results are often quoted in the more recent literature (Alberts *et al.*, 1988; Talcott & Antman, 1988), we are not aware of any other reports on HDMTX therapy of malignant mesothelioma.

Due to the diffuse growth of pattern of pleural mesothelioma, the tumour extension is difficult to evaluate according to classical WHO criteria. We therefore chose to apply tumour thickness as measured by CT scanning as our response parameter, this providing a specific tumour measurement. In most other reports, the method employed for tumour measurement is poorly documented (Colbert *et al.*, 1985; Mintzen *et al.*, 1985; Raghavan *et al.*, 1990; Sorensen *et al.*, 1985). This makes it difficult to directly compare our results with those of others, and it is equally difficult to compare the results of previous studies with each other. The main intention of the present study was thus to investigate whether or not HDMTX is an active regimen in malignant mesothelioma, rather than to compare its activity with that of previously tested agents.

Due to the difficulty in response evaluation in mesothelioma, the effect of treatment on subsequent survival is of particular importance. Median survival in the present series is comparable to that reported in several other series (Alberts *et al.*, 1988; Antman *et al.*, 1988; Brenner *et al.*, 1982), i.e. 11 months for the group as a whole, 12 months for patients with epithelial type, and only 5 months for patients with sarcomatous or mixed types (Figure 3). Some epithelial mesotheliomas are known to have a slow natural growth rate. Studies of untreated patients have thus shown prolonged survival for 10–15% of patients (Law *et al.*, 1984). A slow natural growth rate may also explain why, in our study, stable disease on average lasted longer (median 10 months) than the objective responses (7.5 months). Thirty-two per cent of the patients with the epithelial type survived for more than 2 years, and 18% survived for more than 3 years. This may suggest a possible effect of HDMTX on survival in this histological subgroup. Due to disease progression following HDMTX treatment, 18 patients were subsequently treated with doxorubicin, and nine were treated with palliative radiotherapy. Whether this therapy may have contributed to improved survival remains unknown.

Despite the high age of most patients and the presence of significant amounts of pleural fluid, the treatment with HDMTX did not result in unacceptable toxicity. However, we emphasise that the chemotherapy protocol was followed meticulously, and that the patients were monitored carefully during the MTX excretion phase.

In conclusion, high dose methotrexate is an active regimen in the treatment of patients with diffuse malignant mesothelioma of the pleura, as shown by an objective response rate of 37%. With careful monitoring of the patients, the treatment is safe for patients less than 80 years old in fair condition. Considerable amounts of pleural fluid is not a contraindication to treatment with HDMTX. Due to an indication of short response duration and poor survival in

**Figure 3** Actuarial overall survival for patients treated with HDMTX for malignant pleural mesothelioma, according to histological type. (——), all patients, $n = 63$. (– – –), epithelial type, $n = 42$. (.....), sarcomatous or mixed type, $n = 20$. Time is from the start of HDMTX treatment. The survival difference between patients with epithelial and sarcomatous/mixed types is statistically significant ($P = 0.001$, Log-rank test).

patients with sarcomatous or mixed histological subtypes, the treatment should possibly be reserved for patients with the epithelial type of mesothelioma. However, this finding needs verification in a larger number of patients. It should also be emphasised that the response rate reported in this series should be interpreted with caution, as mesothelioma is a difficult disease to evaluate, necessitating novel methods for tumour measurement. Randomised studies are required to establish whether HDMTX treatment can improve survival for these patients. Considering the cost of HDMTX therapy, the demonstration of a survival benefit seems necessary for the justification of HDMTX as routine treatment for mesothelioma. Finally, the optimal dosage and treatment duration also remain to be established.

### References

AISNER, J. & WIERNIK, P.H. (1981). Chemotherapy in the treatment of malignant mesothelioma. *Sem. Oncol.*, **8**, 335.

ALBERTS, A.S., FALKSON, G., GOEDHALS, L., VOROBIOF, D.A. & VAN DER MERWE, C.A. (1988). Malignant pleural mesothelioma: a disease unaffected by current therapeutic maneuvers. *J. Clin. Oncol.*, **6**, 527.

ANTMAN, K., SHEMIN, R., RYAN, L. & 5 others (1988). Malignant mesothelioma prognostic variables in a registry of 180 patients, the Dana-Faber Cancer Institute and Brigham and Women's Hospital experience over two decades, 1965–1985. *J. Clin. Oncol.*, **6**, 147.

BRENNER, J., SORDILLO, P.P., MAGILL, G.B. & GOLBEY, R.B. (1982). Malignant mesothelioma of the pleura. *Cancer*, **49**, 2431.

BUTCHART, E.G., ASHCROFT, T., BARNSLEY, W.C. & HOLDEN, M.P. (1976). Pleuropneumonectomy in the management of diffuse malignant mesothelioma of the pleura. *Thorax*, **31**, 15.

COLBERT, N., VANNETZEL, J.M., IZRAEL, V. & 8 others (1985). A prospective study of detorubicin in malignant mesothelioma. *Cancer*, **56**, 2170.

DAHL, I.M.S. & LAURENT, T.C. (1988). Concentration of hyaluronan in the serum of untreated cancer patients with special reference to patients with mesothelioma. *Cancer*, **62**, 326.

DAHL, I.M.S., SOLHEIM, ØP., ERIKSTEIN, B. & MÜLLER, E. (1989). A longitudinal study of the hyaluronan level in the serum of patients with malignant mesothelioma under treatment. Hyaluronan is an indicator of progressive disease. *Cancer*, **64**, 68.

DIMITROV, N.V., EGNER, J., BALCUEVA, E. & SUHRLAND, L.G. (1982). High-dose methotrexate with citrovorum factor and vincristine in the treatment of malignant mesothelioma. *Cancer*, **50**, 1245.

FALKSON, G., ALBERTS, A.S. & FALKSON, H.C. (1988). Malignant pleural mesothelioma: the current state of the art. *Cancer Treat. Rev.*, **15**, 231.

LAW, M.R., GREGOR, A., HODSON, M.E., BLOOM, H.J.G. & TURNER-WARWICK, M. (1984). Malignant mesothelioma of the pleura: a study of 52 treated and 64 untreated patients. *Thorax*, **39**, 255.

MINTZEN, D.M., KELSEN, D., FRIMMER, D., HEELAN, R. & GRALLA, R. (1985). Phase II trial of high-dose cisplatin in patients with malignant mesothelioma. *Cancer Treat. Rep.*, **69**, 711.

RAGHAVAN, D., GIANOUTSOS, P., BISHOP, J. & 5 others (1990). Phase II trial of carboplatin in the management of malignant mesothelioma. *J. Clin. Oncol.*, **8**, 151.

SORENSEN, P.G., BACH, F., BORK, E. & HANSEN, H.H. (1985). Randomized trial of doxorubicin versus cyclophosphamide in diffuse malignant pleural mesothelioma. *Cancer Treat. Rep.*, **69**, 1431.

TALCOTT, J.A. & ANTMAN, K.H. (1988). Malignant mesothelioma. In *Textbook of Uncommon Cancer*, William, G.J., Krikorian, J.G., Green, M.R. & Raghavan, D. (eds) p. 309. J. Wiley & Sons Ltd: Oxford.

WHITLEY, N.O. (1987). Computed tomography and malignant mesothelioma. In *Asbestos-related Malignancy*, Antman, K. & Aisner, J. (eds) p. 265. Grune and Stratton Inc: Orlando.